# The Potential of CEB Reinforced Masonry Technology for (Re)construction in the Context of Disasters

**DOI:** 10.3390/ma13173861

**Published:** 2020-09-01

**Authors:** Leandro Di Gregorio, Gustavo Guimarães, Marina Tenório, Daniel Lima, Assed Haddad, Fernando Danziger, Graziella Jannuzzi, Sergio Santos, Silvio Lima

**Affiliations:** 1 Environmental Engineering Program/Urban Engineering Program/Polytechnic School, Federal University of Rio de Janeiro, Fundão Campus, Rio de Janeiro 21941-909, Brazil; assed@poli.ufrj.br (A.H.); jannuzzi@poli.ufrj.br (G.J.); 2 Civil Engineering Department, Federal University of Rio de Janeiro, Macaé Campus, Macaé 27930-560, Brazil; gvmg@poli.ufrj.br; 3 Engineering School, University of Minho Azurém Campus, 4800-058 Guimarães, Portugal; marinaurquiza@poli.ufrj.br (M.T.); daniel.shigue@poli.ufrj.br (D.L.); 4 Civil Engineering Program—COPPE, Polytechnic School, Federal University of Rio de Janeiro, Fundão Campus, Rio de Janeiro 21941-909, Brazil; danziger@coc.ufrj.br; 5 Structural Design Program/Polytechnic School, Federal University of Rio de Janeiro, Fundão Campus, Rio de Janeiro 21941-909, Brazil; sergiohampshire@poli.ufrj.br (S.S.); silvio@poli.ufrj.br (S.L.)

**Keywords:** compressed earth blocks, sustainability, disaster risk reduction

## Abstract

More than 226 million people are affected by some type of disaster every year in various dimensions of human life, both in the short and long term. In this context, housing provision plays a leading role when it comes to basic needs and the choice of construction technologies and materials are determinant for a well-succeeded housing provision process. This work aims to analyze the viability of reinforced masonry technology with cement-stabilized compressed earth blocks as an alternative for the (re)construction process in situations that involve disaster risk reduction (DRR). To address this issue, a discussion from the literature and the main results obtained during the investigations carried out within the scope of the Simple Housing Solution (SHS) Project are presented. In the sequence, analyses are performed under United Nations Development Program/International Recovery Platform sustainability recommendations: environmental, technical, financial and socio-organizational aspects. It is concluded that the technology of Compressed Earth Blocks (CEBs) reinforced structural masonry has a high potential to be successfully applied in DRR situations, especially when associated with the community construction system in a joint effort.

## 1. Introduction

### 1.1. Reinforced Masonry Technology with Cement-Stabilized Earth Blocks

There is no consensus in the literature about the beginning of the earth being used as a building material. Pollock [1] mentions historical records between 5000 and 4000 BC in Mesopotamia, while Berge [2] mentions dates up to 7000 BC, when the use of adobe blocks in Tigris River basins was verified, stating that the earth is one of the oldest building materials employed by man.

Despite the traditional use of earthen building technologies, three centuries ago, it became less attractive than ceramic bricks. In this context, earthen architectures continued to exist only where the scarcity of economic resources required the use of low-cost raw materials for construction [3]. Then, earthen construction assumed a relative connotation of poverty and was even more rejected after the spread of materials related to faster construction processes, such as concrete [4]. However, due to the energetic, environmental, ecological and economic issues raised worldwide in the last decades, a change in mentality has been noticed. This change reframed raw earth as an alternative and sustainable building material dissociated from the idea of discomfort and poverty [4,5,6,7,8]. In the context of earthen construction, Compressed Earth Blocks (CEBs) appear as an evolution of the adobe [9]. This technique consists of pressing a mixture of raw earth and water using manual or automatic, mechanical or hydraulic equipment. Binder materials are usually added to stabilize the mixture. For the purposes of this work, the term CEB will be used to refer to blocks made of a compressed mixture of soil stabilized with cement and water, with the possible addition of hydrated lime.

Regarding the stabilization process, Moreira [10] highlights that binder proportions in a mixture with soil may vary, since soil characteristics significantly change from place to place. Thus, CEB’s quality is not solely dependent on these recommended proportions.

According to Pitta [11], the stabilization with cement and/or lime decreases the earth’s retraction, which is caused by the clay fraction, reducing cracks in blocks after moisture loss. In addition, it increases CEB’s dry unit weight, which improves its behavior in parameters such as compressive strength, thermal properties, tightness and durability, the latter being related to reducing the blocks’ exposure to water. Additionally noteworthy is the fungicidal role of lime in the mixture.

The pressing process allows obtaining modular blocks with regular dimensions, generally with hollows. For the same type of soil, the blocks’ dimensional regularity strongly depends on the production system and on the mixture’s moisture. The use of hand presses causes a significant variability in the manufacturing process, especially in the blocks’ height. For this reason, the use of mortar layers in the construction of CEB masonry is highly recommended, aiming at a better distribution of stresses in masonry elements.

The CEB modular aspect enables a relatively quick and clean construction through blocks interlocking. Thus, architectural design spans should be multiple of ½ block, in order to minimize cuts and waste. In addition, its aesthetic appeal can be explored through the adoption of exposed blocks covered with a thin layer of water-repellent resins or acrylic textures. This protection layer, as well as the waterproof layer on the base of walls, are necessary measures considering that the recurrent action of water on the masonry is a factor that contributes to blocks’ deterioration over time.

To be used in reinforced masonry panels, it is necessary that the CEBs are of a hollow type with two holes. This allows the reinforcement at certain desired points in the masonry. The holes filled with reinforced micro concrete (cement, sand and gravel) add ductility to the structural system, being fundamental in the use of masonry for structural purposes. In addition, the block holes just allow the passage of electrical pipes in a built-in way, since water, sewage or gas pipes are not allowed inside masonry with structural function.

The masonry’s mechanical strength depends, among other factors, on the blocks’ average strength, which should be higher than 2 MPa, according to NBR 8491 (2012) [12]. It is highlighted, however, that the use of CEB masonry for structural purposes is not yet standardized, which motivated the application of the former British Standard BS 5628-2 (2005) [13] (with adaptations from results obtained in laboratory tests) as the basis to structural calculation investigations carried out in this study. It is noteworthy that BS 5628 [13] well-succeeded history of applications and the existence of many constructed buildings in CEB technology allows stating that the use of this material for structural purposes is suitable for buildings with up to two floors, as long as the horizontal loads are not high. Regarding these last cases, which are common in situations involving hazards such as hurricanes and earthquakes, the investigations carried out within the scope of this work indicate that it is possible to obtain disaster-resilient one-story buildings using CEB reinforced masonry, considering horizontal loads up to a certain magnitude.

Studies carried out on the performance of CEBs when used in seismic scenarios show that models constructed using blocks with earthquake-resistant features (sill band, lintel band and vertical bands to control the building vibration) performed better than the models without these reinforcements under dynamic shake table loading on long-period ground motion. Furthermore, models constructed using CEBs performed better than fired brick models.

Herskedal et al. concluded that columns attached to the walls increase the out-of-plane wall stiffness significantly [14] and, in locations of high seismicity, a limit of 3.0 m between aligned columns may be necessary to reduce damage [15]. Despite that, the contribution of a reinforced concrete confining frame increased the total strength with about 20% relative to the bare wall [16]. Bland [17] suggests by his tests that CEB shear strength is not directly proportional to the net area, and is attributed to a more continuous distribution of grouting, and better “shear continuity”.

Srisanthi et al. state that the earthquake-resistant characteristics can prevent the collapse of off-plan walls of one or two-story buildings in a strong earthquake if the proper monolithic behavior of mooring columns and masonry walls is achieved. Thus, single-story confined masonry buildings, properly designed and constructed, could be used in high seismic zones [18]. Stirling [19] suggested by his results from the incremental dynamic analysis on single-story demonstration buildings that there is a relatively low probability of earthquake-induced collapse for similar single-story flexure dominant CEB constructions.

Srisanthi et al. [18] also emphasize the importance of developing manuals and training to enable construction in CEB suitable for seismic situations.

### 1.2. The Context of Disasters

“Disasters” are understood as the consequences of an adverse event (a phenomenon caused by man and/or nature) in a vulnerable environment, which exceeds the response capacity of the affected social system. These consequences are represented by human, material and environmental damages and their consequent socioeconomic and patrimonial losses. Thus, the word disaster does not refer to the phenomenon itself (flood, hurricane, etc.), but to the adverse effects caused in the affected ecosystem. The disaster’s harmful effects are directly proportional to vulnerability and exposure of the elements at risk in its various aspects: physical, environmental, economic, political, organizational, institutional, educational and cultural [20].

According to the UN, more than 226 million people are affected by some type of disaster every year [21] and it is estimated that, in 2017 alone, the related financial losses reached about 330 billion dollars [22]. Natural disasters bring about 26 million people to poverty every year [23]. According to the EM-DAT database [24], in the last decade, approximately 120 thousand deaths and 8.87 million displaced people were registered worldwide due to natural disasters. These numbers refer to events such as volcanic activity, earthquakes, storms, tidal waves, landslides, floods and forest fires that directly affected more than 855 million people since 2011. In the last year alone, 353 thousand displaced or homeless people were counted worldwide.

Disasters have an impact on many dimensions of human life, both in the short and long term. In this context, housing provision plays a leading role when it comes to basic needs. In all disasters, the loss of housing is the second biggest concern, coming after the loss of lives [25]. UN-Habitat [26] estimates that near 100 million people are currently homeless and one in four live in conditions that are harmful to safety and health. In addition, it foresees for 2030 the need for adequate housing for about 40% of the world population, which means a demand for 96,000 new housing units per day.

Losses and damages from geological threats have grown rapidly, reaching around 15 thousand victims per year, which is not due to the increase in seismic activity, but rather to the increase in vulnerable settlements [27]. Blondet points out that, in developing countries, most people live in nonengineered low-rise constructions made of poor materials, which makes them more vulnerable [28].

In this scenario, the need for a housing provision system geared towards disaster risk reduction (applied on a large scale) is evident, especially for the most vulnerable. United Nations Development Program (UNDP) and International Recovery Platform (IRP) [29] highlight that the most important is that the housing solution should be sustainable and present five key principles for this to happen:

- Environmental sustainability. The chosen approach should avoid the depredation of natural resources and contamination of the environment;
- Technical sustainability. The required skills can be introduced and taught to others, and the necessary tools are available;
- Financial sustainability. Money or exchange of services can be used to pay for the service that needs to be executed;
- Organizational sustainability. There must be a structure to aggregate different actors without the need to involve outside experts in each situation;
- Social sustainability. The final process and product should meet the expectations and needs of society.

Construction technologies and materials play an important role in the housing provision process, being closely linked to the level of acceptance of the new homes. The influence increases even more when it comes to an application in the ODR/CDR (owner-driven/community-driven reconstruction) systems, where the participation of residents in the process is high and the community’s cultural aspects tend to be more present in the solutions adopted. However, it is worth mentioning that local building materials and technologies should be employed to the extent that they are consistent with disaster risk reduction.

Based on the recommendations of UNDP and IRP [29], the aim of this work is to analyze the viability of reinforced masonry technology with cement-stabilized compressed earth blocks as an alternative in the (re)construction process in situations that involve disaster risks. Thus, it may be useful to the guidance of technical teams in the process of choosing constructive technology to be adopted for housing provision in situations of disaster risk reduction, especially when resources are scarce. To address this issue, a discussion will be held from the literature and the main results obtained during the investigations carried out within the scope of the Simple Housing Solution (SHS) Project will be presented.

## 2. SHS Methodology

**Figure 1:**
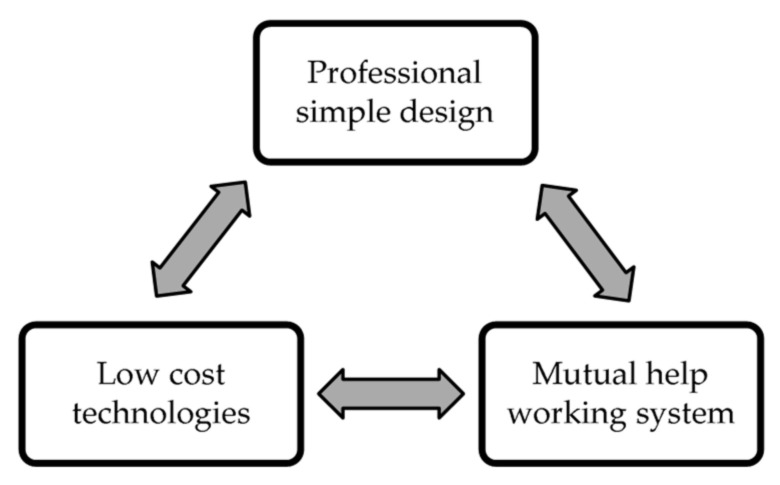
Simple Housing Solution (SHS) methodology tripod.

The SHS methodology started with the SHS Technological Innovation Project (2009–2012) and was improved along with the SHS Extension Project at Federal University of Rio de Janeiro (UFRJ) (2017–2019), both coordinated by the first author of this paper. The methodology aims to provide knowledge that may potentially facilitate the process of (re)construction in critical situations. It was conceived with the philosophy of gathering basic knowledge that can be useful in the (re)construction of housing units and basic collective equipment (schools and health clinics), in a joint effort (community construction) and using low-cost technologies, constituting the tripod illustrated in Figure 1. The idea is to contribute so that disaster-affected communities can better organize themselves for their own recovery, counting on the guidance and supervision of qualified technical assistants (engineers and/or architects) to be hired by the community itself, government or NGOs.

SHS Project’s efforts were directed to study the technology of CEB reinforced masonry for structural purposes, as it understands that this technology allows high correlation with the three axes on the tripod shown in Figure 1.

SHS buildings were designed based on a modular coordination by ½ block module, using as reference a block with approximate dimensions of 25 cm × 12.5 cm × 6.5 cm (length × width × height), which can be manufactured by a mechanical hand press. This way of fabrication was prioritized in the project, due to its simplicity and to the fact that it does not need electricity, being aligned with disaster contexts where the availability of resources is reduced. In addition, technical characteristics of blocks manufactured in this way tend to be lower than those manufactured with automatic machines, which allows the assessment of technical feasibility in unfavorable conditions.

**Figure 2:**
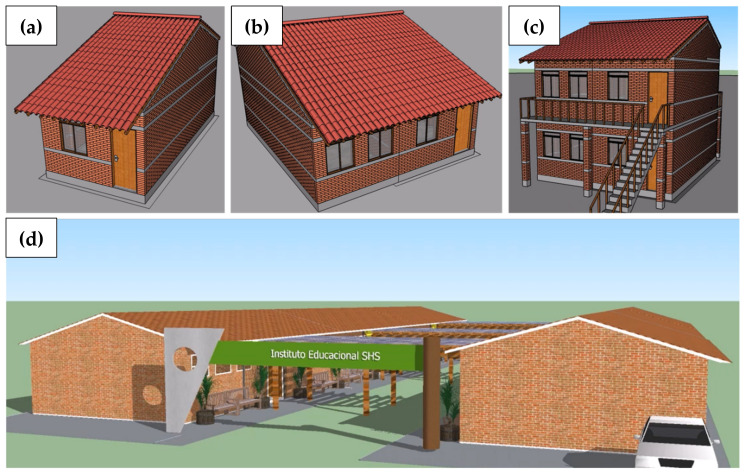
Models for situations with usual horizontal loads. (**a**) Embryo 1; (**b**) Embryo 2; (**c**) Embryo 4; (**d**) School [31].

In all, four residence models, six options for school modules and two options for health clinics were designed to be combined in the way considered most appropriate by the community (Figure 2). The residences start from a basic model with 24 m^2^ (Embryo 1), which seeks to meet the basic shelter needs for a family of four. From Embryo 1, horizontal enlargement (Embryo 2) and later vertical enlargement (Embryo 4) can be performed, comprising up to two families of six people each. Embryo 3 corresponds to a direct vertical enlargement from Embryo 1, but this model was not considered a priority.

The SHS methodology’s teaching material consists of slides, video classes, electronic spreadsheets and drawings related to the construction of low-cost popular houses, organization of community joint efforts and the administration of construction works, which must be evaluated and adapted, case by case, by those who wish to implement them. A YouTube channel (PROJETO SHS) was also launched, in which 30 video classes are available [30].

The detailed project’s history, the generated products and their phases can be consulted directly on the website [31], which is available in Portuguese (PT), English (EN), Spanish (ES), French (FR) and Haitian Creole (CR). SHS Project received an honorable mention from the international UN Sasakawa Award 2019, ranking among 16 finalists.

The situations related to the context of disasters are those that aim to reduce the risk, as described in the following cases:

- Case A: Situations that involve the relocation of people from risk areas (predisaster). Even though the disaster has not happened yet, the aim is to relocate vulnerable populations that are currently at risk in the places where they live, moving those to safer areas. Ex.: situations involving areas at risk of flooding, landslides, dam failure, etc.
- Case B: Situations for reconstruction in another location (postdisaster). This case would be applicable where the disaster has already occurred, but the present site is considered to be a risk area. Ex.: situations that involve floods, landslides, dam failure, etc.
- Case C: Situations for reconstruction onsite (postdisaster). In this case, the disaster has already occurred, but it is considered that there is no significant risk reduction in the relocation process and that vulnerability is more related to the houses where people live. Ex.: earthquake situations, strong winds, etc.

For disaster situations that do not involve high horizontal loads (Cases A and B), residences with up to two floors (Embryos 1, 2 and 4) proved to be adequate, as long as some care is taken to conserve the residences and their masonry, especially against prolonged humidity. If the masonry is exposed to water dragging forces, it is recommended to use coatings with greater mechanical resistance, such as mortar or ceramic tiles.

However, for disaster contexts where horizontal loads are high (included in Case C above), the models initially developed in SHS Project (Embryos 1–4) do not apply. This is basically due to the following reasons:

- It might have a complete integration between masonry panels, so that vertical joints are not desirable. Thus, the construction of Embryo 1 for further expansion to Embryo 2 is not possible;
- The vertical expansion from Embryos 2–4, or from Embryos 1–3, is not feasible, as the bending moments at the base become very high.
- The arrangement of the openings in Embryos 1, 2 and 4 favors a collective arrangement of residences that minimizes the use of the land, allowing the residences to be built together (there are no openings in lateral walls). This results in an asymmetric openings’ distribution, which is not suitable for dynamic loads.

**Figure 3:**
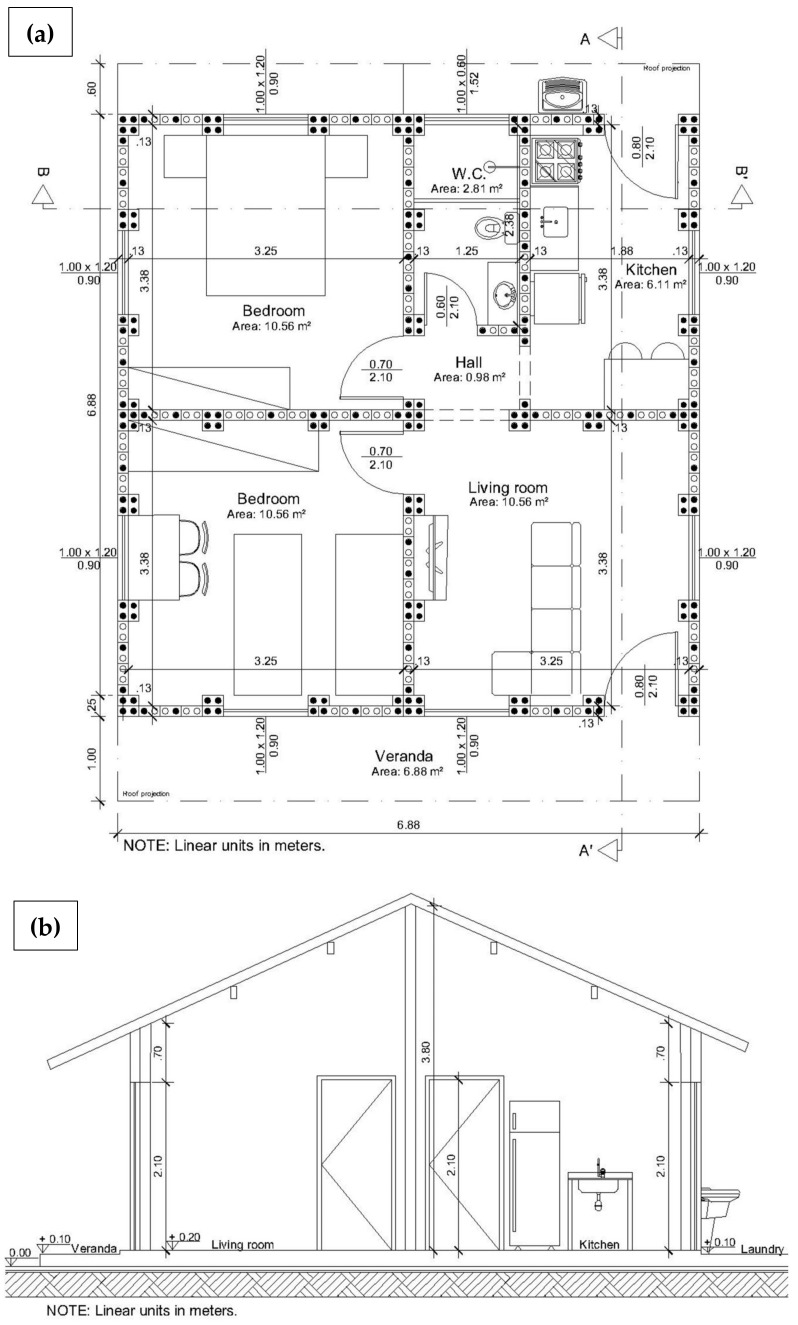
(**a**) Embryo 2C floor plan; (**b**) Section AA’ [34].

**Figure 4:**
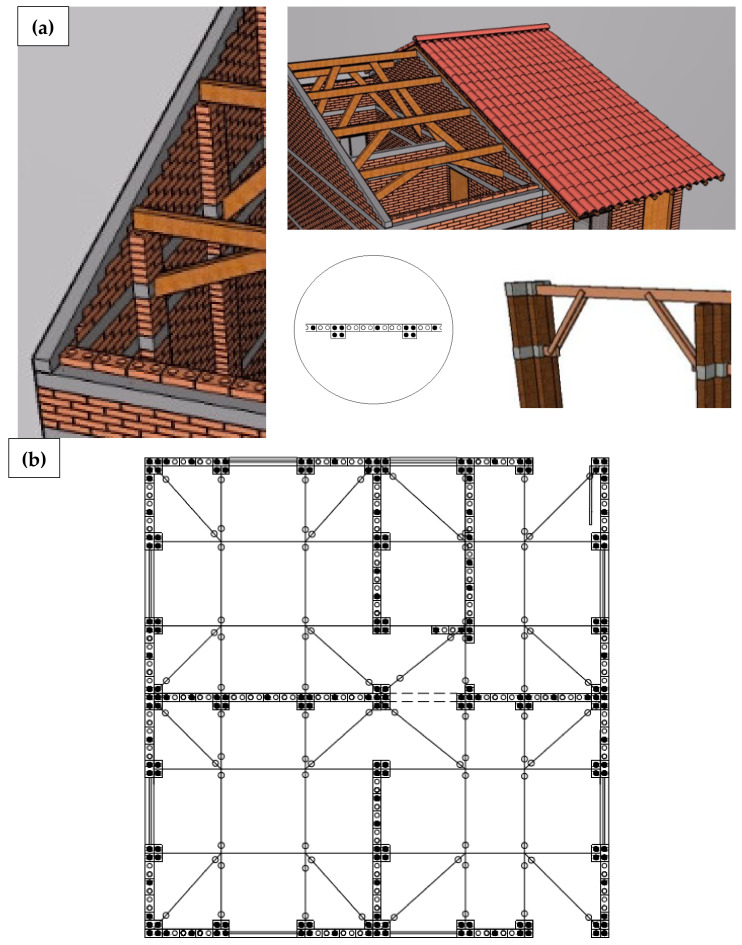
(**a**) Portico system [31]; (**b**) Embryo 2C roof structure [34].

To meet the situations of Case C, SHS Project developed a residence model with only one floor, slightly modified from Embryo 2, and which does not allow lateral or vertical enlargements (Figure 3). This model has more favorable characteristics to withstand dynamic horizontal loads within a certain range of magnitude, such as:

- Absence of slab;
- Reduced ceiling height;
- Roof gables with symmetrical planes;
- Relatively symmetric distribution of openings;
- Position of stiffeners on the sides of doors and windows openings;
- Use of the roof structure integrated into the structural scheme of the house (Figure 4b);
- Adoption of a portico system that integrates walls and roof structures, common to Cases A, B and C (Figure 4a).

It is noteworthy, however, that there are intrinsic limitations to CEB reinforced masonry technology, which restricts the range of action in relation to hazards such as earthquakes and strong winds.

Embryo 2C’s design was supported by results obtained during the SHS experimental campaign. Regarding CEB masonry’s structural behavior, tests on wallets and columns were carried out aiming to obtain mechanical properties and wallet-block efficiency. Horizontal load capacity tests (adapted shear tests) were performed to get inputs for computational models used to investigate the SHS Embryo 2C residential model under seismic conditions, according to NBR 15421 (2006) [32]. The estimate of wind loads was made according to NBR 6123 (1988) [33]. Structural verifications were based on BS 5628-2 (2005) [13]. These points will be detailed in Section 3 and Section 4.

## 3. Masonry Components’ Properties

### 3.1. Materials and Methods

To carry out laboratory tests and the manufacture of CEBs over the years 2017–2019, two minifactories were set up at Rio de Janeiro State, Brazil: one at Polytechnic School of UFRJ (located at the Sustainable Materials and Technologies Lab, NUMATS POLI/COPPE) and another at UFRJ/Macaé Campus. Various tests were conducted at these facilities to determine properties of the soils used in block’s manufacture, as well as postmanufacture tests. Two deposits of unsaturated tropical soil (Soils 1 and 2), located at UFRJ/Macaé Campus, were investigated and a geotechnical characterization was carried out in the laboratory.

**Figure 5:**
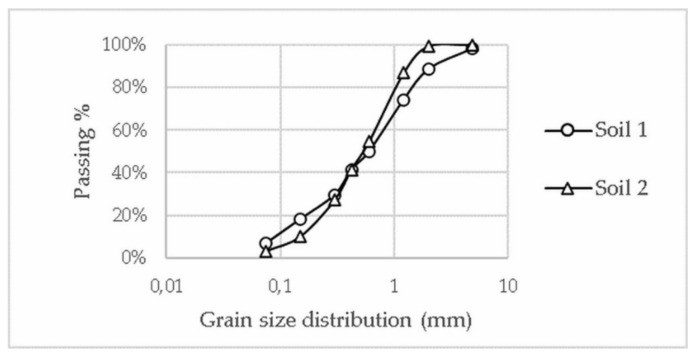
Grain size distribution graph of samples from Soils 1 and 2 [39].

Soil samples were prepared and tested according to International Standards. Soil preparation, grain size distribution, specific gravity and Atterberg limits were obtained following ISO 23909 (2008) [35], ISO 17892-4 (2016) [36], ISO 17892-3 (2015) [37] and ISO 17892-12 (2018) [38], respectively. An empirical test was also carried out to verify soil retraction during the natural drying process. Grain size distribution graphs of both soils are presented in Figure 5 and CEBs’ production was carried out with the portion with a grain size smaller than 5 mm.

#### 3.1.1. Tests Performed for Obtaining Wallet-Block Efficiency

##### Blocks’ Compressive Strength Tests

**Figure 6:**
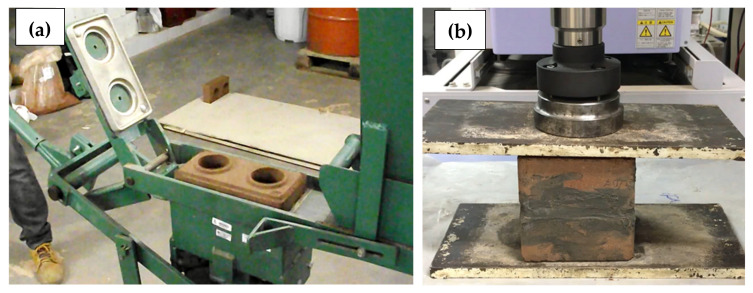
(**a**) Compressed Earth Block’s (CEB’s) manufacture process using a mechanical hand press; (**b**) CEB’s compressive strength test.

CEBs were manufactured using a mechanical hand press (Figure 6a), from a mixture of 50% Soil 1 and 50% Soil 2, in volume, here called S1S2. The blocks used cement as a binder with different volume proportions in the ratio 1:X (1 part of cement to X parts of mixed soil). The final mixtures used 2% of lime and water content next to 20%. Indeed, the soil moisture was empirically controlled in a tactile way, aiming to bring laboratory conditions closer to outer conditions in a project application situation, where the community itself would do this task. It should be noted that water content can significantly vary within different types of soils. CEBs’ water absorption and compressive strength were verified according to NBR 8492 (2012) [40], but using samples described in Table 1, Table 2 and Table 3. Figure 6b shows the CEB’s compressive strength tests carried out at NUMATS, in a Shimadzu hydraulic press with a maximum capacity of 100 kN and at a load rate of 500 N/s.

##### First Wallet’s Compressive Strength Tests

To obtain the wallet-block efficiency used in structural verifications, they were carried out compressive strength tests (adapted from NBR 16522, 2016 [41]) on three wallets with a length of 1 m (equivalent to four blocks per row), a clear width of 10.5 cm and a height next to 70 cm (equivalent to the elevation of 10 rows of blocks). The blocks used to build the wallets were produced under a 1:8 (cement: soil) proportion and their age exceeded 28 d. All three specimens had their blocks laid in a ~1 cm thick industrial mortar (the same used for tiling) joint, manually mixed. The grout was manually mixed using a 1:3:2 volume proportion (cement, sand, 3/8″ stone gravel) and a water/cement ratio of one. The water/cement ratio was chosen based on the understanding that, in practice, communities would probably add a high amount of water to the grout, aiming to facilitate the work. Two symmetrical 50 cm spaced holes were grouted and reinforced with 1/4″ CA 50 steel bars (one per hole). At the top, a capping mortar layer composed of cement and sand in a 1:3 proportion regularized the face that received the load. The wallets’ compressive strength tests were carried out at NUMATS after 7 d, in a WPM hydraulic press with a maximum capacity of 3000 kN and at a load rate of 500 N/s.

#### 3.1.2. Tests Performed for Obtaining Inputs to Seismic Analysis

##### Shear Test

In shear walls subjected to inplane shear, a mechanism of failure of the oblique strut may be generated [42]. To investigate this issue horizontal load capacity (shear) tests were designed aiming at the principle of portraying in the specimens the boundary conditions of a wall panel confined between two reinforced concrete joists and two consecutive reinforced columns, in order to obtain the mechanical properties in that quadrant. The test consists of the application of a horizontal load on a wallet with a length of 1 m (equivalent to four blocks per row), a clear width of 10.5 cm and a height next to 1 m (equivalent to the elevation of 13 rows of blocks and a concrete joist at the top). The blocks used to build the wallets were produced under a 1:6:0.5 (cement–soil–basalt powder) proportion and their age was greater than 28 d [43]. All three specimens had their blocks laid in a ~1 cm thick mortar joint, manually mixed at a volume proportion of 1:1:6 (cement, lime, sand). The grout was manually mixed using a 1:6:4 volume proportion (cement, sand, 3/8″ stone gravel) and a water/cement ratio of 1. The reinforcement of holes and joists counted on 1/4″ CA 50 steel bars (one per hole and two per joist).

**Figure 7:**
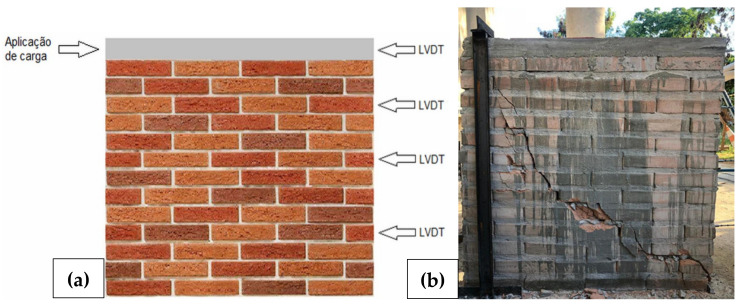
(**a**) Design of horizontal load tests; (**b**) Final stage with the cracked wall [34].

The reinforced concrete joist had the same width as the blocks and reflected the upper confinement of the panel. Vertically, the wall had its lateral holes reinforced and grouted. In addition to these, two more intermediate holes were symmetrically reinforced and grouted, representing panels with two reinforced holes per meter. To avoid rotation, a steel support embedded to the concrete basis was added next to the load application point (but with no translational restrictions in the direction of the applied load) and, to avoid global translation in the direction of the applied load, a steel angle was added at the bottom of the wall, at the load point’s opposite corner (Figure 7b).

The load was manually increased and applied by a hydraulic jack positioned in the middle of the joist’s section. The load stages were 150 kg on average. Due to the manual process, the load rates could not be controlled, but in order to stabilize displacements the waiting time for each stage was about 30 s. The displacements were measured with LVDT (linear variable differential transformer) at four different levels (LVDTs 1, 2, 3, 4 from the top to the bottom). A load cell was used to monitor the load. Displacement and load were acquired at the same time using a data acquisition system. Figure 7a shows the design of the horizontal tests and Figure 7b shows one of the cracked wallets.

##### Second Wallet’s Compressive Strength Tests

**Figure 8:**
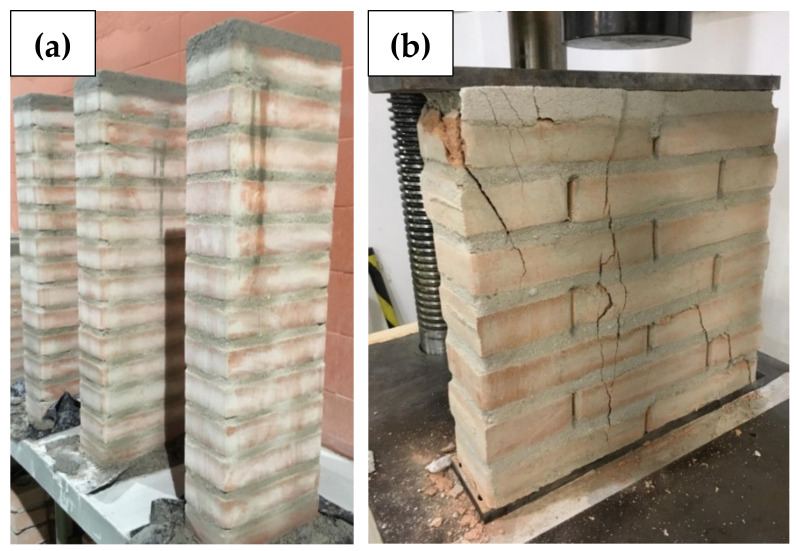
(**a**) Specimens for axial compression tests (fully reinforced small columns); (**b**) Axial compression test carried on a 50 cm length wallet with one reinforced hole [34].

To obtain the elastic modulus used in the computational seismic model, wallet compression tests were performed following the recommendations of the British Standard BS 1052 (1999) [44] (Figure 8b). It recommends that, for blocks with dimensions of 25.0 cm × 12.5 cm × 6.5 cm, the specimens must have a minimum of 50 cm length and height greater than or equal to the length, but limited to 1.875 m. The wallet specimens were built using the same materials applied to the specimens described in the shear test, with two blocks in length (equivalent to 50 cm in length and 10.5 cm in width) and 50 cm high, totalizing seven rows laid on mortar joints. To simulate the effect of grouted columns arranged every 50 cm in the proposed structure, the joint near the central hole was reinforced and grouted. Mechanical properties obtained in wallet compression tests were used in the compressed diagonal bars at Embryo 2C’s computational model, as detailed in Section 4.1.

The columns’ specimens (Figure 8a) had one brick per row, resulting in a clear length of 23 cm and a clear width of 10.5 cm. With an elevation of 13 rows laid on mortar joints, the average height of the specimens was 90 cm, which fits the position of the contour concrete middle joist. On those specimens, all two holes were reinforced and grouted. Mechanical properties obtained in columns compression tests were used in the vertical bars at Embryo 2C’s computational model, as detailed in Section 4.1.

The wallet’s and column’s specimens were tested 21 d after built-in hydraulic presses at a load rate of 500 N/s.

### 3.2. Results

#### 3.2.1. Tests Performed for Obtaining Wallet-Block Efficiency

##### Blocks Compressive Strength Tests

It was found that the blocks with a 1:8 volume proportion reached a mean compressive strength higher than 2 MPa, a value higher than the minimum established by NBR 8491 (2012) [12] for the production of CEBs. Table 2 and Table 3 show the results obtained with the quantities of specimens according to the volume proportion, the average values (μ) of the compressive strength of CEBs and the standard deviation (σ).

**Figure 9:**
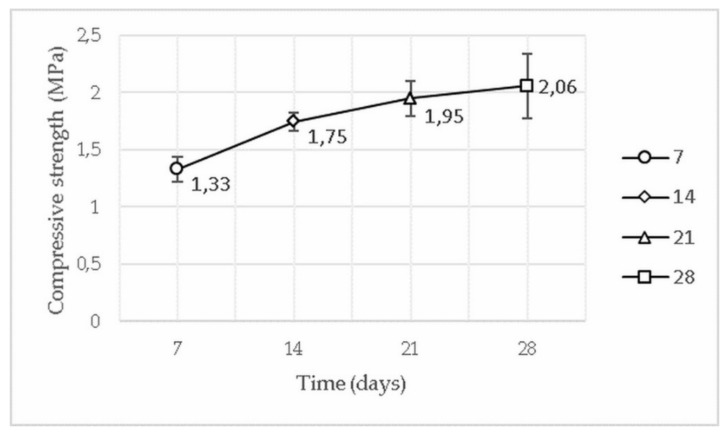
Evolution of the resistance gain of CEBs with 1:8 volume proportions [39].

From the tests performed, an increase in compressive strength was observed following the increase in cement proportion; however, it was not possible to establish a growth pattern since the manual manufacturing process, the material’s heterogeneity and the circumstances of curing and humidity control implied a considerable dispersion on the results. The mean resistance value obtained for the 1:6 mix was even lower than the 1:8 mix, either due to the factors mentioned or due to the high content of powdery material compared to the amount of water in the mixture, justified by the greater amount of cement in the 1:6 mix, which leads to a superior powdery material content when comparing to other volume proportions. In addition, it was noted, through tests performed at 7, 14, 21 and 28 days after production, an increase in the compressive strength of the 1:8 blocks (Figure 9) in proportions similar to that of cement, verifying the greatest resistance gain in the first seven days.

##### First Wallet’s Compressive Strength Tests

Regarding the behavior of masonry, wallets compressive strengths tests were carried out, preceded by the characterization of its components. The grout and mortar resistances used are shown, respectively, in Table 4 and Table 5 below.

The results for the wallets compressive strength test were obtained (Table 6).

For wallet-block efficiency, defined by the ratio between the mean resistances of wallets and blocks, a value of 0.59 was obtained (Table 7). Considering that wall-wallet efficiency for ceramic blocks is estimated at about 85% [45] and assuming the same proportion may apply to CEB masonry, one can estimate the CEB wall-block efficiency as 85% × 0.59 = 0.50. This result is considered coherent, since the values related to wall-block efficiency for concrete blocks vary between 0.4 and 0.6, according to Ramalho and Corrêa [46]. However, it should be noted that the efficiency obtained is related to wallets (dimension 1 m × 0.7 m) in CEB masonry panels reinforced by 50 cm spaced grouted columns.

#### 3.2.2. Tests Performed for Obtaining Inputs to Seismic Analysis

##### Shear Test

**Figure 10:**
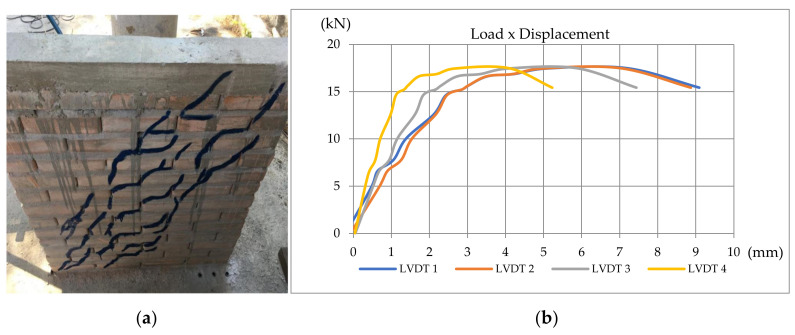
(**a**) Specimen 1; (**b**) Load–displacement curve for Specimen 1 [34].

**Figure 11:**
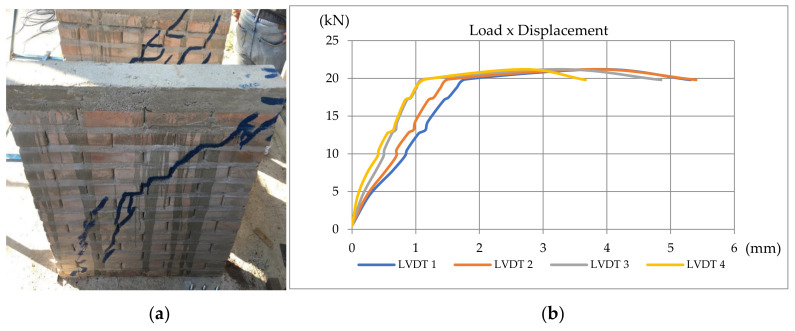
(**a**) Specimen 2; (**b**) Load–displacement curve for Specimen 2 [34].

**Figure 12:**
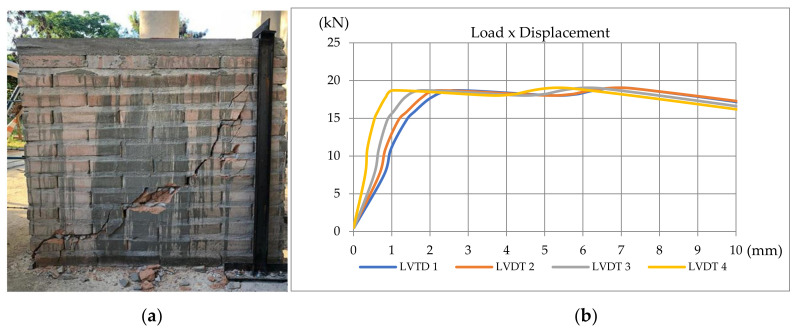
(**a**) Specimen 3; (**b**) Load–displacement curve for Specimen 3 [34].

During the shear tests, it was noticed that the rupture occurs in the compressed diagonal, given the tensile forces imposed on the opposite diagonal, resultant from the shear. From the applied load and the measured displacements, they obtained the results presented in Figure 10, Figure 11 and Figure 12.

##### Second Wallet’s Compressive Strength Tests

**Figure 13:**
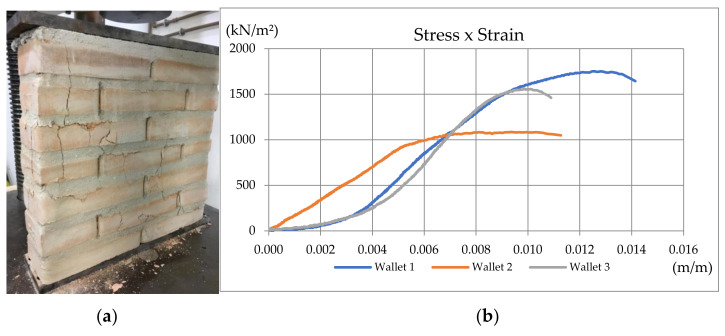
(**a**) Wallet Specimen 1; (**b**) Stress–strain curve for 50 cm spaced grouted wallet specimens [34].

**Figure 14:**
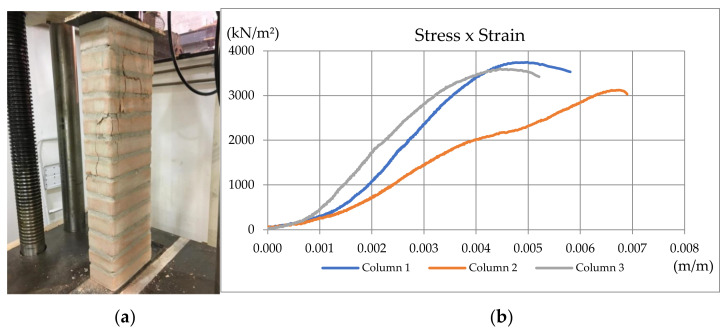
(**a**) Column Specimen 1; (**b**) Stress–strain curve for fully grouted column specimens [34].

In addition, compression tests were performed on wallets and columns to determine their elastic modulus. From these tests, the following graphs, shown in Figure 13 and Figure 14, were obtained.

Regarding the construction of shear test’s specimens, grout and mortar compressive test results are listed in Table 8 and Table 9.

For the 50 cm spaced grouted wallets, the elastic modulus was obtained from the average of the elastic modulus present in Table 10, but without the results from Wallet 2, which was considered an outlier. All were obtained at the linear part of the stress–strain curves, delimited by Points P1 and P2. Thus, the elastic modulus for the wallets showed μ = 259,824 kN/m^2^ and σ = 45,551 kN/m^2^.

For the fully grouted columns, the elastic modulus was obtained from the average of the elastic modulus presented in Table 11, but without the results from Column 2, which was considered an outlier. All were obtained at the linear part of the stress–strain curves, delimited by Points P1 and P2. Thus, the elastic modulus for the grouted columns showed μ = 1,204,008 kN/m^2^ and σ = 395,008 kN/m^2^.

The elastic modulus and specific gravity attributed to other materials (wood) were obtained from the literature.

## 4. Seismic Analysis

### 4.1. Materials and Methods

The seismic analysis was performed by an application of the Equivalent Horizontal Force Method, according to the prescriptions of the Brazilian seismic standard NBR 15421 (2006) [32], in a linear static analysis. Depending on the earthquake-resistant structural system, NBR 15421 defines the following coefficients: response modification (R), overstrength factor ($\Omega_{0}$) and deflection amplification factor ($C_{d}$), which are used to determine the forces and displacements. These coefficients allow the transformation of elastic response spectra into design response spectra, in which ductility is considered. This way, through a linear static analysis, it is possible to address the structure’s nonlinear behavior. To carry on the Equivalent Horizontal Force analysis, the following parameters were defined:

- Use category: I—Usual structures;
- Structure importance factor: I=1.0;
- Basic earthquake-resistant system: porticos that resist at least 25% of seismic forces and masonry walls with common reinforcement (ASCE 7-05);
- Response modification coefficient R=3.0;
- Overstrength factor $\Omega_{0}=3.0$;
- Deflection amplification factor $C_{d}=2.5$.
- Irregularities in the plan: None;
- Vertical irregularity: None.

#### 4.1.1. Computational Model

**Figure 15:**
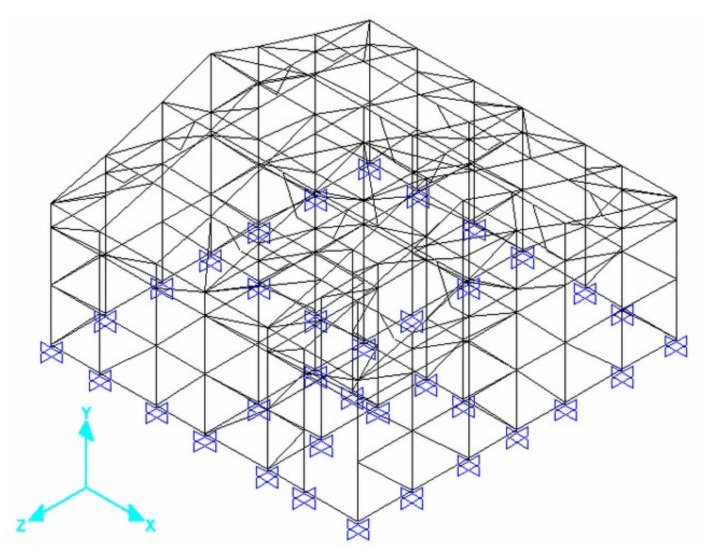
SHS Embryo 2C’s computational model [34].

An initial 3D frame (Figure 15) computational model was built on the basis of SHS Embryo 2C’s architectural design, whose vertical bars represent the columns embedded at the basis and the horizontal bars represent the concrete joists in three levels, besides the gable top joist. Roof elements were also represented as bars and wood beams linked to the walls to integrate the portico system. Rigid connections between columns and joists were considered.

**Figure 16:**
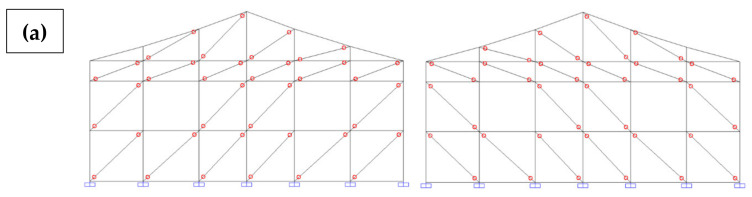

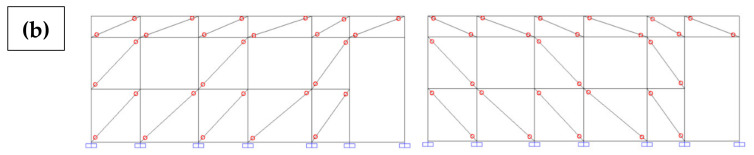
Options for compressed diagonal bars. (**a**) Lateral facade; (**b**) Front facade [34].

Therefore, the masonry panels confined between the columns and joists were represented as diagonal bars and were considered only when compressed. To this end, the model was initially designed with two diagonals on each masonry panel. A horizontal load was imposed in each of the four directions separately and, through the results of this analysis, it was possible to determine and eliminate the tensile bars for each situation (Figure 16).

Diagonal bars were characterized according to experimental data obtained for the elastic modulus. The horizontal load test results were used to calibrate the cross-section of these bars (supposed squared) in the computational modeling of an equivalent wall panel. This panel model is composed of horizontal bars, which represent the upper and lower confinement of the masonry, and vertical bars, which represent the lateral confinements. 

**Figure 17:**
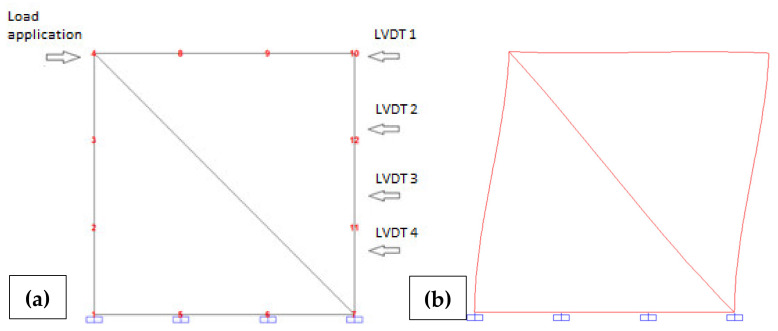
(**a**) Panel model used to calibrate the diagonal bar; (**b**) Deformed shape of the panel used for the diagonal bar’s calibration [34].

The mean maximum lateral load obtained in the experiments (19.23 kN) was then imposed at the top of the model (Node 4) and the squared section for the diagonal bar was calibrated, aiming that the average displacement obtained in Node 10 after the static analysis (6.3 mm) is the same as the observed at LVDT 1 (Figure 17a), resulting in the panel deformed shape correspondent to the diagonal bar’s calibration (Figure 17b). Therefore, for equivalent panels of dimensions 1 m × 1 m, a cross-section of 10.5 cm × 12.1 cm (width × height) for diagonal bars was adopted.

It is important to highlight that the elastic modulus adopted for all wall panels was obtained in the linear part of the stress–strain diagrams (Table 10). However, as we did not consider the lower value of three the elastic modulus to calculate the average, the panels’ stiffness might be overestimated, which would restrict displacements and raise the house frequency, against security. This way, we opted to calibrate the panels in the nonlinear part of the force–displacement curve, in a reduced stiffness configuration, by reducing the equivalent cross-section. Conservatively, this criterium allowed larger displacements, resulting in larger efforts at the basis of the columns (which stiffness was kept in the elastic regime), lowering the house frequency and keeping the response spectral pseudo acceleration at the top of the design response spectrum for different load scenarios, which guarantees maximum seismic loads, as long as maximum horizontal displacements are obtained. It is also worth mentioning that structural verifications on walls were carried out considering sections composed of real panels/stiffeners and were not based on the diagonal bars equivalent section.

#### 4.1.2. Load Scenarios

**Figure 18:**
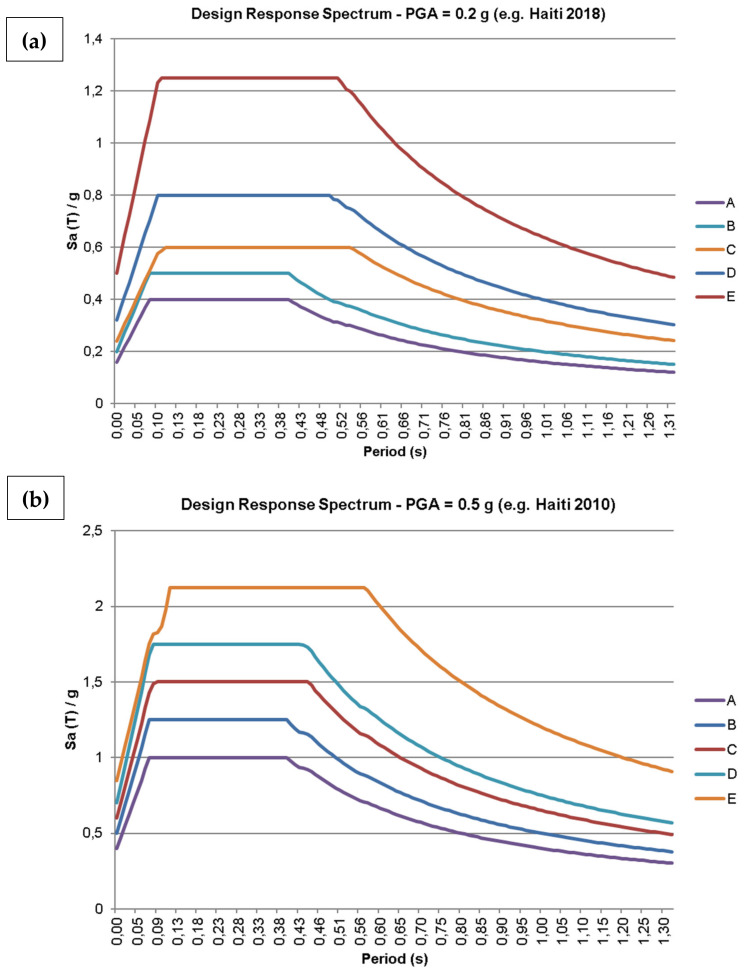
(**a**) Design response spectrum for PGA = 0.2 g (e.g., Haiti, 2018); (**b**) Design response spectrum for PGA = 0.5 g (e.g., Haiti, 2010) [34].

For the seismic analysis, which was based in the Equivalent Horizontal Force method, two different earthquake scenarios were chosen: light (PGA = 0.20 g) and moderate (PGA = 0.50 g) earthquakes, each one combined with five types of foundation soils defined by the Brazilian seismic standard NBR 15421 (2006) [32]: A—healthy rock, B—rock, C—altered rock or very rigid soil, D—rigid soil and E—soft soil. This resulted in 10 different scenarios represented by the design response spectra in Figure 18, where $S_{a}\left(T\right)$ is the pseudo acceleration response spectrum, *g* is the gravity acceleration and T is the natural vibration period, in seconds, associated with each of the structure’s vibration modes.

When applying the Equivalent Horizontal Force method, the total horizontal force at the base of the structure is evaluated, in a given direction, according to the expression $H=C_{s}\cdot W$, where W represents the total structure weight corresponding to permanent loads, and $C_{s}$ is the seismic response coefficient, defined as $C_{s}=\frac{2.5\frac{a_{gs0}}{g}}{\frac{R}{I}}$. I is the importance factor corresponding to the use of the structure. The seismic response coefficient is limited by the expression $0.01\leq C_{s}\leq \frac{\frac{a_{gs1}}{g}}{T\cdot \left(\frac{R}{I}\right)}$. $a_{gs0}$ and $a_{gs1}$ correspond to the spectral acceleration for the period of 0.0 s and 1.0 s, respectively. The approximate period of the structure is given by the expression $T_{a}=C_{T}\cdot h_{n}^{x}$, where $h_{n}$ represents the height of the structure above the base, $C_{t}=0.0488s/m$ e x=0.75 (period coefficients for other structures, given that the basic earthquake-resistant system is composed of frames and masonry walls). The fundamental period of the structure was also obtained through a modal extraction process using SALT UFRJ software.

**Figure 19:**
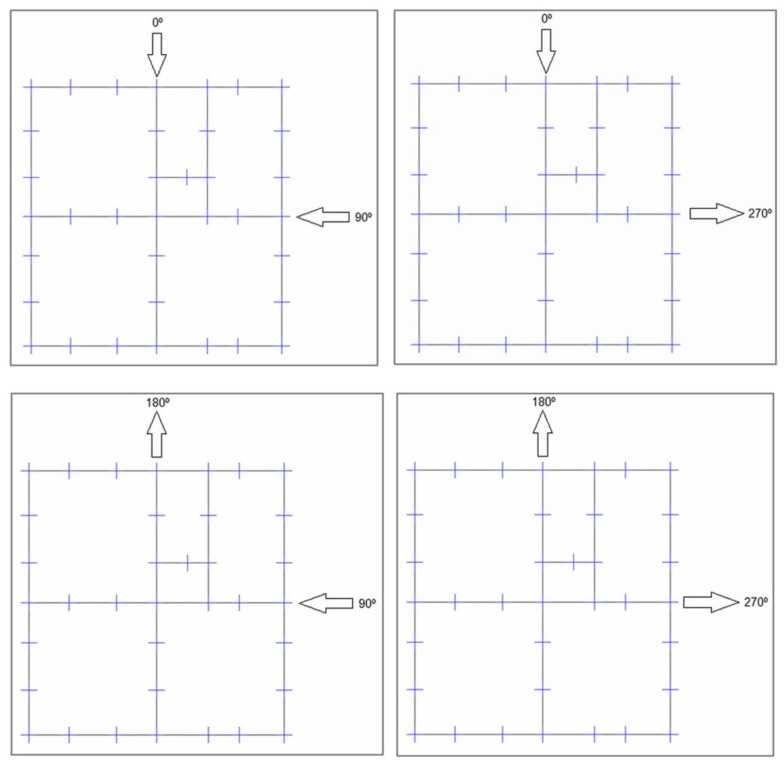
The four load application configurations in SHS Embryo 2C’s footprints [34].

Aiming at considering different load directions, four different models were generated (Figure 19). To each were applied the configurations with compressed diagonal bars, as previously discussed. An accidental torsional moment (torque) was considered due to a displacement of the mass center, in each direction, equal to 5% of the dimension of the structure located perpendicular to the seismic horizontal forces.

Considering the load combinations as appointed in NBR 15421 (2006) [32], one can have the following load application scenarios (that imply eight different models):

- 0 degree: 100% Earthquake 0° + 30% Earthquake 90° + 100% Torque 0°;
- 90 degrees: 100% Earthquake 90° + 30% Earthquake 90° + 100% Torque 90°;
- 0 degree: 100% Earthquake 0° + 30% Earthquake 270° + 100% Torque 0°;
- 270 degrees: 100% Earthquake 270° + 30% Earthquake 0° + 100% Torque 270°;
- 180 degrees: 100% Earthquake 180° + 30% Earthquake 90° + 100% Torque 180°;
- 90 degrees: 100% Earthquake 90° + 30% Earthquake 180° + 100% Torque 90°;
- 180 degrees: 100% Earthquake 180° + 30% Earthquake 270° + 100% Torque 180°;
- 270 degrees: 100% Earthquake 270° + 30% Earthquake 180° + 100% Torque 270°.

**Figure 20:**
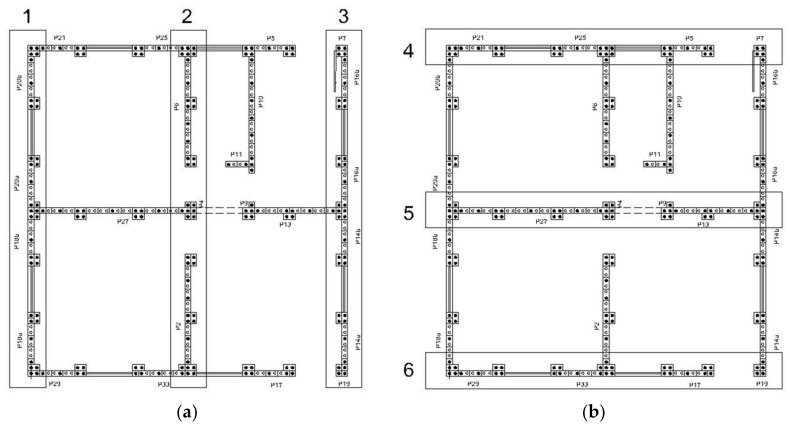
(**a**) Panels in the 0°/180° direction (Figure 19); (**b**) Panels in the 90°/270° direction (Figure 19) [34].

The total seismic load attributed to the walls, calculated according to the Equivalent Horizontal Force method, was divided between the shear wall panels located in horizontal axes (Figure 20), according to their masses (Table 12), and was uniformly distributed along the panels, at the lintel level. The same was done for the roof, which had its load distributed between the roof’s purlins according to their area of influence. To consider accidental torsional moments, a torque was applied on the extreme walls, with opposite signal forces and distributed along the lintel.

#### 4.1.3. Structural Verifications

**Figure 21:**
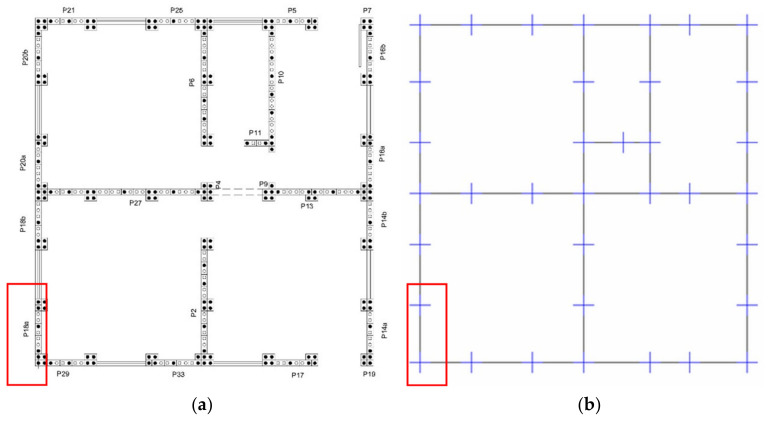
(**a**) Isolated walls used in structural verifications; (**b**) Correspondent computational basis nodes at the columns’ basis [34].

After running the model on the SALT UFRJ software, the bending moments and forces obtained at the column’s basis were transferred to the near system columns/panels, in order to proceed with structural verifications (Figure 21). This is because all the verifications carried out are done wall by wall, making it convenient to determine the nodes that contribute to their respective walls. For nodes shared between transverse walls, it was admitted that the moment and forces in the shared node would be absorbed only by the walls that were aligned to the direction of the effort. 

Forces and bending moments obtained from the computational analysis were combined according to NBR 8681 (2004) [47] using the equation $E_{d}=1.2E_{g}+1.0E_{q}+1.0E_{exc}$, where $E_{d}$, $E_{g}$, $E_{q}$ e $E_{exc}$ are, respectively, the total combined action and the partial actions due to permanent, accidental and seismic loads.

From the most unfavorable efforts obtained for each panel, structural verifications were carried on the wall sections composed of the system wall panels/stiffeners for the ultimate limit state. These verifications were based on BS 5628-2 (2005) [13] with adaptations when necessary, and considered walls as isolated, ignoring the group effect between and providing a conservative approach. Thus, for each wall, the following verifications were carried out:

- Bending (in wall plane, outside the plane and oblique);
- Resistant vertical load (axial compression, compression combined with bending, axial tensile);
- Horizontal shear parallel to the joints.

In addition, according to the method of equivalent horizontal force, the largest displacements must be verified. The absolute displacements $\delta_{x}$, evaluated at the center of mass of the structure, are determined by the expression $\delta_{x}=\frac{C_{d}\times \delta_{xe}}{I}$, where $\delta_{xe}$ represents the displacements determined in static analysis, considering the application of the forces $F_{x}$; $C_{d}$ is the displacement amplification coefficient and I is the structure importance factor.

**Figure 22:**
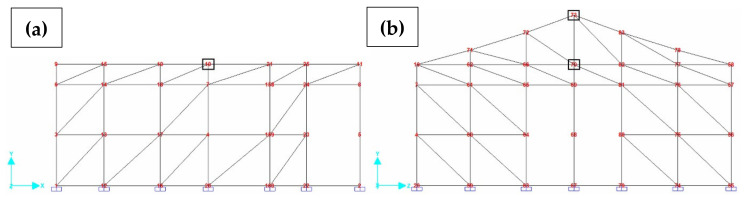
The 3 nodes used for the analysis of the maximum displacements of the undamaged and damage structure. (**a**) Front and back facades. (**b**) Lateral facades [34].

The maximum displacements obtained at three different points were analyzed (for the maximum Equivalent Horizontal Force supported by the structure): Nodes 10, 70 and 73 (Figure 22). Node 10 (Figure 22a) represents the displacements transverse to the wall plane performed by the front and back walls (on the *z*-axis), Node 70 (Figure 22b) represents the out-of-plane displacements at the lintel level (on the *x*-axis) and Node 73 (Figure 22b) represents the out-of-plane displacements at the highest point of the structure, on the roof gable (on the *x*-axis). In the present case (one-story building), absolute and relative displacements for each node are the same and are limited to $0.020\;h_{sx}$, where $h_{sx}$ is the distance between the node and the basis.

### 4.2. Results

The total calculated weight of the structure was 309.69 kN (64.47 kN for the roof and 245.22 kN for the walls). Starting from this, for each load scenario, they were calculated equivalent horizontal forces (Table 13).

For Equivalent Horizontal Force scenarios equal to or greater than 103.23 kN, failures are noted mainly due to shear stresses on the wall plane, which is the determining factor in the design of this structure when under seismic loads. Therefore, Table 13 shows the percentages for wall lengths approved for horizontal shear, disregarding the results of bending and resistant vertical load, whose decrease of approved walls were very small from one scenario to another.

It was concluded that Embryo 2C in CEB structural masonry can resist earthquakes of up to 0.20 g PGA (with a horizontal force of up to 82.58 kN) in Class A, B, C or D terrains, in a near collapse limit state analysis. This acceleration is similar to the earthquake that happened in Haiti in 2018.

The approximate period of the structure is $T_{a}=0.0488\cdot {\left(3.77\right)}_{0.75}=0.13s$ and the fundamental period obtained from the modal extraction is near 0.15 s. These similar values reveal that the computational model reflects the expected behavior for single-story buildings. One can note that the period obtained from modal extraction is slightly greater than the approximate period, perhaps due to the reduced stiffness adopted for the wall panels. However, this criterion led to more unfavorable displacements, as explained before. Despite that, for the displacement analysis carried out for the structure under earthquakes up to 0.20 g, it was concluded that the displacements maximized by the coefficient $C_{D}$ are significantly lower than those limited by NBR 15421 (2006; Table 14).

Regarding the study related to the effects of wind action on the structure, it was concluded that (in a structure similar to the proposal but with different openings distribution) it would be able to withstand forces corresponding to winds with speeds up to 35 m/s (126 km/h) [48] since some reinforcements are made and the roof tiles are released (fuse effect).

## 5. Discussion

The sustainability of the proposed housing solution will be assessed according to the five key principles listed by UNDP and IRP [29]: environmental sustainability, technical sustainability, financial sustainability, organizational sustainability and social sustainability.

### 5.1. Environmental Sustainability

In the context of high environmental impacts of civil construction, an increase in studies of vernacular techniques is noted, such as the use of earth as a building material, which, according to studies such as [6,49,50], can reduce the associated environmental impacts, especially with regard to the incorporated energy and carbon dioxide emissions. Morton [51], for example, considers the possibility of this type of construction reusing part of the residual soils produced by the construction industry in the United Kingdom (about 24 million tons per year).

During the phases of materials production and construction, which include activities of extraction of raw material, refinement, manufacturing, transportation and construction process, the use of CEBs has a great potential to reduce the impacts. Although the earth is not a renewable resource, its extraction for the production of CEBs, since it can be carried out by manual means, mitigates the environmental impacts related to this activity. In addition, considering the use of local soil (for the production of blocks and subsequent construction) reduces the impacts related to transportation, which, according to Berge [2], represents a major contribution to the low environmental performance of construction materials. The CEBs manufacturing method is also an ally in reducing CO and CO_2_ emissions to the atmosphere, since it is based only on pressing the material, making the firing process unnecessary for curing the block, which eliminates the stage of burning wood or fuel [52]. This process can be further benefited if hand presses are used instead of automatic ones.

Lourenço [53] developed a comparative study of energy expenditures related to the production phase that considers several construction systems. The results show that the prototype in CEB bearing masonry, crowned with concrete lintels and covered with wooden beams, presented less than half of the energy consumption performed by the prototype in reinforced concrete structure, ceramic pierced brick masonry and roof slab in prefabricated joists and tiles. In another study, Maza [54] achieved results that show that 1 m^2^ of wall in traditional ceramic brick reaches an impact associated with the life cycle five times greater than that obtained in 1 m^2^ of wall in CEB stabilized with cement and lime, in an analysis developed in Ecoindicator 99. Part of these results are due to the fact that CEB masonry allows the suppression or reduction of several conventional construction processes, mainly the use of wooden forms, mortar coverings and breaking of masonry for the passage of installations.

With regard to the CEB’s end of life, it is understood that earth-based construction materials can simply be returned to the extraction site, without any environmental risk. Even if lime or cement was added to stabilize the soil, there is still the possibility of reusing wasted or demolished material for the manufacture of new blocks and the construction of new buildings [9]. Therefore, it can be considered that the use of CEBs practically does not generate waste, except when associated with other types of materials and construction systems [9].

CEB construction also has environmental benefits associated with indoor air quality, since its aesthetic character allows, in certain cases, the exposure of walls without any coating, which prevents, for example, high releases of VOCs by paints based on organic solvents; and it has the ability to control relative humidity by absorption (up to 10 times higher than traditional ceramic brick constructions [55]) while maintaining indoor humidity at intervals considered appropriate for human health [51]. However, the need to apply water-repellent resin or acrylic texture is emphasized, especially on the external face of external walls and bathrooms, in addition to waterproofing the masonry base, in order to avoid recurring contact with humidity and increase the system’s life. Shower booth walls and washable areas should be waterproofed.

However, Fernandes et al. [56] highlight the lack of studies that allow the precise quantification of the environmental impacts associated with the earth use at the expense of conventional materials, according to the applicable rules in force. This fact can be justified by the heterogeneity of the soil properties according to the extraction site, its small-scale production and the consequent difficulty in normalizing its use.

### 5.2. Technical Sustainability

UN-HABITAT [57] points out that earthen and stone buildings have good thermal inertia and a lot of potential in the low-cost housing sector, with approximately ⅕ of the world population living in adobe or clay constructions. New ways of producing and using compressed earth blocks, for example using stabilized soil technologies, have improved the skills of traditional adobe blocks, making them more attractive technically and economically. According to the author, materials based on earth or stone are, in general, recyclable with low environmental impact, have good heat and sound insulation capabilities, are fast and economical to build, are natural, healthy and nonflammable.

Throughout SHS Project’s work, an attempt was made to approach manufacturing conditions in a situation of real application, where the community itself would be responsible for the manufacture of the bricks. In this way, the production process of the blocks was carried out with a mechanical hand press and tactile humidity control by volunteer undergraduate students who did not have previous practice in this type of activity.

Among all the cement–soil volume proportions tested (1:6, 1:8, 1:10, 1:12), CEBs with a 1:8 mix presented the best results in compressive strength, with a mean above 2 MPa, the minimum permitted by technical standards. It is noteworthy that the results show high dispersion, which is assumed to be related to one or more of the following factors: the quality and heterogeneity of the soil, variations in the compaction moisture of the mixture, the curing process, the material content powdery, variations in the pressing force, variations in the regulation of the press, among others. More detailed analyses were performed by the SHS team in [39,43,58].

Analyzing the evolution of the compressive strength over time, it was concluded that, despite the largest portion of resistance gain occurring in the first seven days, it is essential to ensure adequate cure during the 28 days for greater safety and quality of the CEBs, recommending its use only after the end of this period.

Regarding the use of CEB as a material with a structural function, the tests carried out showed that the block-wallet efficiency obtained is within the expected for blocks for this purpose, but attention should be paid to the materials chosen for the composition of grout and mortar, in order to guarantee the good compatibility of deformations between the materials, a fundamental aspect for structural systems in reinforced masonry.

For the dynamic load scenarios evaluated, the importance of compatibility between materials is highlighted in order to guarantee the expected performance of the masonry, in addition to the good execution of connections between walls, foundations and roof, as they represent the most relevant points for the force distribution and where the first ruptures are usually verified. The most suitable type of foundation in areas with potential soil liquefaction is still under study. Additionally, the need for reliable low-cost soil investigation methods in that case is recognized.

From the analysis developed for the structure when affected by dynamic horizontal load phenomena, namely earthquakes, it was possible to verify that such resistant behavior is only possible due to the masonry reinforcement. In the present case, if there were no steel elements, responsible for checking the structure’s ductility, the seismic resistance would be seriously affected, even for 0.20 g PGA, because, according to the verifications carried out, the walls would go into a rupture process due to the out-of-plane bending forces. More detailed analyses were performed in [34].

Other studies carried out within the scope of the SHS Project have investigated the feasibility of arrangements for the distribution of residences onsite [59,60] and the viability of building a mini-CEBs factory within the construction site [61]. Regarding this last aspect, it was concluded that the minifactory installation proves to be more advantageous from projects with more than 60 housing units.

It was found that the main challenge for the minifactory installation is the restriction of physical space, which imposes limitations on the blocks’ storage until the conclusion of the 28-day curing period and could require efficient transport logistics. Regarding the laboratory, it was concluded that the main difficulty associated with its implementation at the construction site is related to the availability of financial resources for the acquisition of equipment, in addition to the need for qualified professionals to carry out the tests [61]. Aspects related to occupational health and safety in the activities of the housing construction project were also investigated in [62].

### 5.3. Financial Sustainability

When analyzed separately, CEB reinforced masonry can be considered more expensive than a conventional masonry with reinforced concrete structure, traditionally used in Latin American construction.

However, when considering the full cost of construction, the savings can be significant. This is because the construction system in CEB reinforced masonry practically eliminates the use of wooden forms, as well as the need for mortar coating. Costs with rips in masonry for the passage of electrical pipes are also practically eliminated, as well as the use of built-in pipes, since the holes in the blocks carry them as conduits, as long as they are not filled with grout.

Although, when compared to the structural masonry construction system in concrete blocks, the costs of both systems are shown to be close, as they suppress the same expenses in construction [63].

Studies carried out under SHS Project show that, when analyzing the direct costs of labor, materials and equipment for the construction of the Embryo 2, the economy of CEB reinforced the masonry system with purchased blocks, compared to a conventional system, was nearly 14%. If the blocks are manufactured at the construction site, the savings can reach 23% (Table 15).

However, another factor can contribute even more significantly to reducing costs. This is the production system with the use of labor from the community itself, on a voluntary basis (joint effort or community construction). In this system, families are organized into work groups, so that everyone works for the collective enterprise. It should be noted that this system differs from the self-construction system, where each family builds its own house. Therefore, due to the way the teams are organized, the collective construction system favors the use of specialized teams that perform their tasks repeatedly in several houses, while the self-construction system is more suitable for the use of multitasking teams, since the family works permanently in the place of their own home.

At this point, once again the technology of CEB reinforced masonry plays a decisive role, as it is simple enough to be assimilated and dominated by unskilled labor, typical of collective construction enterprises, and that it employs preferably locally available materials, reducing logistics costs.

For these reasons, the community building system has several characteristics aligned with the Lean Construction philosophy, an adaptation of the Toyota Production System (TPS) for civil construction and based on the optimization of time, cost and consumption of the project’s resources. Among these characteristics, the following stand out: a structure based on the repetition of activities, the organization of tasks in clearly defined work packages, the organization of specialized teams with relative autonomy of action and self-control, the definition of a similar chained work rhythm between teams, all in the vicinity of a “pulled” production system, that is, where the successor activities are triggered from the demand of the completed predecessor activities.

It can be seen, from the cost comparisons in Table 12, that the economic benefits of the construction technology plus the application of a community construction system can lead to savings of up to 55% in the direct cost of the house. It should be noted, however, that the costs of contracting, administration of the task force, assembly of the CEB factory and other indirect costs are not considered here. Besides that, it should be clarified that community members, even though trained, do not meet the need for professionals specialized in leading the tasks of the working groups. Thus, if the communities do not have these professionals among their volunteer members, they will have to be hired, reducing the economy of the community construction system.

In terms of economic and time feasibility, it was demonstrated that the construction method is the one that determines the cost and, mainly, the term of the work, concluding that labor savings can reach almost 33% when using CEB structural masonry instead of conventional masonry of ceramic bricks. Furthermore, it is confirmed that the association between the construction method using CEBs and the community construction system may be responsible for reducing up to 50% of the cost of houses built using SHS methodology [65].

### 5.4. Socio-Organizational Sustainability

The role of building materials and technology is fundamental for the community construction system to be adopted; however, it is far from being sufficient.

The collective construction approach requires a high level of involvement and cooperation on the part of the beneficiaries, but IFRC and RCS [66] warn that it is important not to idealize the notion of “community”, which may not be cooperative on all issues. In addition, Barakat [67] adverts that in order to be successful, cooperative reconstruction must be carefully organized and administered, which requires certain managerial and technical capacity on the part of the agencies involved in the implementation. Relationships between the community and implementing agencies must be good and construction objectives and goals must be established before any construction work can begin. Jha et al. [68] specify that the community-led recovery approach is useful where:

- New construction technologies, materials or house designs are being introduced;
- Agencies provide construction materials;
- House reconstruction is linked to community development activities.

In a survey carried out with 20 joint effort enterprises located in 15 Brazilian municipalities, the Habitare Project [69] reveal that costs are approximately 30% lower than the conventional process, due to the following factors:

- Reduction in direct labor expenses;
- No financial charges or profit;
- Reduced costs for food, transportation, expenses for the central office and construction site;
- The careful purchase of materials, when made by the community, also contributes to cost reduction and buildings quality assurance [70].

Especially in recoveries that actively involve the participation of homeowners, authors surveyed mentioned the improvement in the sense of self-organization of the affected group and the feeling of belonging/identification/satisfaction with the final product. In this way, it is reiterated that housing recovery provides a broad development field, in which the value lies not only in the product, but mainly in the process.

To verify the interest of the affected population in participating in a rebuilding process in a joint effort, they were conducted field interviews with homeless families in the municipality of São José do Vale do Rio Preto, RJ, Brazil, after the megadisaster in the mountainous region of Rio de Janeiro in January 2011, when it was found that 90% of respondents would accept to participate in an owner/community-led recovery approach, even involving the use of voluntary labor by residents and others [71]. This result is attributed to the urgency and insecurity imposed by the homelessness situation, as well as to the culture of self-construction/task force present in most low-income Brazilian communities.

Other studies carried out within the SHS Project’s scope have shown similar results in different contexts. From a survey conducted in the context of the Don de L’Amitié neighborhood, Haiti, hit by Hurricane Matthew in October 2016, it was indicated that the entire population interviewed was interested in the process of rebuilding their community from a joint effort [72]. In a survey conducted with residents of areas at risk of flooding in the municipality of Barra Mansa, RJ, Brazil, an index of 85% of adherence was obtained to the proposal for construction in a joint effort [59].

It is understood, therefore, from a socio-organizational point of view, that SHS methodology in CEB reinforced masonry is a promising option to be applied in most disaster contexts as an alternative in the recovery portfolio.

## 6. Conclusions

The construction technology plays a central role in the (re)construction process in disaster contexts, with CEB reinforced masonry being considered promising for use in these situations.

To confirm this hypothesis, discussions were presented on several aspects of this technology, based on the UNPD and IRP sustainability recommendations [29]:

- Environmental sustainability, where the use of local materials, simple production and recyclability were the main highlights;
- Technical sustainability, where results of several tests and analyses demonstrated the strength of the structural system for the proposed buildings aimed at light horizontal loads (Embryos 1–4) to moderate (Embryo 2C) horizontal loads;
- Financial sustainability, where it has been shown that, when associated with joint efforts, the savings generated can reach up to 50% compared to the traditional Latin American construction system;
- Socio-organizational sustainability, which sought to highlight the interest of the affected population and the benefits of the technology application in a joint effort for the community organization.

The seismic approach brought by the article aimed to discuss the potential of CEB reinforced masonry technology for seismic situations without exhausting the subject. For this reason, new tests are being proposed for the next phase of the SHS Project, when cyclic tests on porticos and full-scale dynamic tests on shaking tables are intended to be performed.

Regarding the context of disasters (reconstruction with relocation, reconstruction on the spot, or relocation of people living in risk areas) or simply in contexts where resources are scarce and the reduction of the housing deficit for the most vulnerable populations is desired, it is concluded that the technology of CEB reinforced structural masonry has a high potential to be successfully applied, especially if associated with the community construction work system in a joint effort.

## Figures and Tables

**Table 1:** Components of CEBs.

Soil Type	Binder	Proportion (Cement–Soil)	Water Content	Age (days)
S1S2	Cement	1:6, 1:8, 1:10 and 1:12 (+2% of lime)	Variable according to the soil moisture	7, 14, 21 and 28

**Table 2:** Compressive strengths of the tested CEBs with a 1:8 volume proportion [39].

CEB	μ (MPa)	σ
S1S2–1:8–7 days	1.33	0.12
S1S2–1:8–14 days	1.75	0.07
S1S2–1:8–21 days	1.95	0.15
S1S2–1:8–28 days	2.06	0.28

**Table 3:** Ratio of volume proportions, quantities and compressive strengths of the tested CEBs [39].

Volume Proportion	Quantity	μ (MPa): 28 days	σ
1:6	3	1.92	0.08
1:8	3	2.06	0.28
1:10	11	1.38	0.15
1:12	4	1.34	0.22

**Table 4:** Grout compressive strength test results [39].

Grout (Wet Curing)	Quantity	μ (MPa)	σ
1:3:2 (cement, sand, gravel)–28 days	6	6.34	0.3

**Table 5:** Mortar compressive strength test results [39].

Mortar	Quantity	μ (MPa)	σ
Prefabricated Mortar–7 days	4	4.44	0.23

**Table 6:** Wallet’s compressive strength test results [39].

Wallets	Quantity	μ (MPa)	σ
S1S2–1:8–28 days	3	1.22	0.24

**Table 7:** Strength parameters of CEB partially reinforced masonry components [39].

Element	Compression Strength (MPa)
Block	2.6
Grout	6.34
Mortar	4.44
Wallets	1.22
η (dimensionless)	0.59

**Table 8:** Grout compressive strength test results [39].

Grout (Dry Curing)	Quantity	μ (MPa)	σ
1:6:4 (cement, sand, gravel)—14 days	3	1.46	0.05

**Table 9:** Mortar compressive strength test results [39].

Mortar	Quantity	μ (MPa)	σ
1:1:6 (cement, lime, sand)—14 days	3	3.46	0.16

**Table 10:** Elastic modulus for the wallets, obtained from the linear part of stress–strain curves between Points P1 and P2 [34].

Wallet	Coordinates (Strain; Stress)		Elastic Modulus (kN/m^2^)
	P1	P2	
1	(0.00392; 289.56533)	(0.00807; 1314.67276)	246,683
2	(0.00170; 287.15219)	(0.00430; 767.01448)	184,281
3	(0.00419; 287.21124)	(0.00795; 1314.65390)	272,966

**Table 11:** Elastic modulus for the grouted columns, obtained from the linear part of stress–strain curves between Points P1 and P2 [34].

Column	Coordinates (Strain; Stress)		Elastic Modulus (kN/m^2^)
	P1	P2	
1	(0.00179; 857.79462)	(0.00340; 2862.93209)	1,243,479
2	(0.00222; 860.54865)	(0.00605; 2868.22443)	523,258
3	(0.00133; 857.82402)	(0.00305; 2865.49938)	1,164,537

**Table 12:** Mass distribution along the shear panels illustrated in Figure 20 [34].

Panels in 0°/180° Direction	% Mass of the Structure	Panels in 90°/270° Direction	% Mass of the Structure
1	17.20%	4	13.25%
2	19.39%	5	19.71%
3	17.20%	6	13.25%

**Table 13:** Seismic scenarios and the evaluation for horizontal shear efforts, parallel to the joints. Adapted from [34].

PGA	Class of Foundation Soil	$S_{a}$	$C_{s}$	H—Equivalent Horizontal Force (kN)	Approved Masonry for Horizontal Shear Parallel to the Joints
0.20 g e.g., Haiti 2018	A	0.40 g	0.133	41.29	100%
	B	0.50 g	0.167	51.62	100%
	C	0.60 g	0.200	61.94	100%
	D	0.80 g	0.267	82.58	100%
	E	1.25 g	0.417	129.04	79.81%
0.50 g e.g., Haiti 2010	A	1.00 g	0.333	103.23	91.29%
	B	1.25 g	0.417	129.04	79.81%
	C	1.50 g	0.500	134.85	55.98%
	D	1.75 g	0.583	180.65	31.27%

**Table 14:** Displacements verification for PGA = 0.5 g soil class A (more aggressive than scenario PGA = 0.2 g soil class D). Adapted from [34].

Node	$h_{sx}$ (cm)	$\delta_{x}$_lim (cm)	$\delta_{xe}$ (cm)	$\delta_{x}$ (cm)	Verification
	270	5.4	0.38	0.96	OK
70	270	5.4	0.40	1.01	OK
73	380	7.6	0.45	1.12	OK

**Table 15:** Comparison of costs between conventional system and CEB reinforced masonry, having as reference the residential model Embryo 2 with 61.92 m^2^ [64].

Criteria	Conventional Masonry	Purchased CEBs	Manufactured CEBs
**Typology**	Conventional masonry + fiber cement roof	Purchased CEBs + acrylic texture sealing (external walls)	CEBs manufactured onsite + acrylic texture sealing (external walls)
**Total Cost**	BRL 87,471.60	BRL 75,385.49 (−14% *)	BRL 67,527.92 (−23% *)
**Cost Without Labor**	BRL 51,780.66	BRL 47,338.44 (−46% *)	BRL 39,480.87 (−55% *)
**With Septic Tank-Filter System**	BRL 54,316.52	BRL 49,874.31	BRL 42,016.74
**With Septic Tank-Filter System + Rainwater sse**	BRL 56,212.03	BRL 51,769.81	BRL 43,912.74
**Cost per m^2^**	BRL 1,412.66	BRL 764.51	BRL 637.61

Indirect costs and land not included. Prices May 2019, SINAP RJ. * Reference.
